# Total immunoglobulin E levels in induced sputum reflect asthma control status

**DOI:** 10.1002/clt2.70021

**Published:** 2025-01-05

**Authors:** Wenhui Chen, Xiaofang Liu, Xiujuan Yao, Yanghe Hao, Zhuo Zhou, Chengshuo Wang, Ming Wang, Luo Zhang

**Affiliations:** ^1^ Department of Otolaryngology Head and Neck Surgery Beijing TongRen Hospital Capital Medical University Beijing China; ^2^ Beijing Key Laboratory of New Medicine and Diagnostic Technology Research for Nasal Diseases Beijing Laboratory of Allergic Diseases Beijing Institute of Otolaryngology Beijing China; ^3^ Department of Respiratory and Critical Care Medicine Beijing Tongren Hospital Capital Medical University Beijing China; ^4^ Department of Allergy Beijing TongRen Hospital Capital Medical University Beijing China

**Keywords:** asthma control, immunoglobulin E, induced sputum, uncontrolled asthma

## Abstract

**Background:**

Most patients with severe asthma are sensitized to at least one allergen. Whether local immunoglobulin E (IgE) in induced sputum reflects asthma control status has not been investigated.

**Methods:**

Patients with asthma were classified as well controlled, partly controlled, and uncontrolled asthma (UCA) according to Global Initiative for Asthma 2022 guidelines. Lung function and fractional exhaled nitric oxide (FeNO) were evaluated. Induced sputum was collected and total IgE and Phadiatop (IgE to common inhalant allergens) measurements were performed. General clinical characteristics and pulmonary inflammation indicators were analyzed between the three groups of asthmatic patients. Univariate and multifactor ordinal logistic regression were used to model the relationship between pulmonary inflammation indicators and asthma control status. The ability of sputum total IgE in identifying different levels of asthma control was assessed by receiver operating characteristic curve (ROC).

**Results:**

Patients with UCA had worse lung function and airway inflammation as indicated by lower levels of forced expiratory volume in 1s (FEV1)%pred, FEV1/FVC, MEF75%pred, MEF50%pred and MEF25%pred, and higher levels of FeNO and sputum eosinophil% compared with the WCA group. In addition, higher levels of total sputum IgE and Phadiatop were found in patients with UCA than in patients with WCA and PCA. Univariate and multifactor ordinal logistic regression analysis indicated that sputum total IgE was the unique significant risk factor for poor asthma control (adjusted odds ratio = 6.25; 95% CI, 1.07–36.55; *p* < 0.05) among pulmonary inflammation indicators including different indices of pulmonary function test, sputum IgE and FeNO. Sputum total IgE levels showed a significant correlation with asthma control scores (*r* = 0.53, *p* < 0.001). Moreover, ROC analysis showed that the predictive value of sputum total IgE for patients with UCA was 0.82 (95% CI, 0.74–0.9).

**Conclusion:**

Sputum total IgE reflects levels of asthma control, and can be used as an indicator of UCA.

## 1 INTRODUCTION

Asthma is a heterogeneous chronic inflammation of the airway that affects around 339 million people worldwide, with approximately 5%–10% of the patients suffering from severe or uncontrolled asthma (UCA).^1^ Typical manifestations of asthma include coughing, wheezing, chest tightness and shortness of breath, which substantially impact patients' quality of life.^2^ According to the Global Initiative for Asthma (GINA) guideline, achieving good symptom control is a long‐term goal in asthma management.^3^ In particularly, poorly controlled asthma not only greatly impacts quality of life, but also creates economic burdens for individuals and society.^4^ According to the GINA guideline, levels of asthma control are defined as well controlled (WCA), partly controlled (PCA), and UCA. The precise assessment of asthma control levels is essential for instructing the clinic practice. However, the reported indicators that can predict levels of asthma control are very limited.

Levels of asthma control are associated with pulmonary local indicators, such as fractional exhaled nitric oxide (FeNO) and forced expiratory volume in 1s (FEV1), which discriminate between different control levels. However, clinical effectiveness of FeNO or FEV1 in monitoring asthma control level is still debated.^5^, ^6^, ^7^, ^8^ Epidemiological studies have shown that atopic sensitization is a strong risk factor for asthma in both childhood and adulthood.^9^ Although the role of allergy might be more important in the pathogenesis of severe asthma in early life, studies in adults have shown that three quarters or more patients with severe asthma display sensitivity to at least one allergen.^10^, ^11^, ^12^ To date, there is no evidence on the relationship between local immunoglobulin E (IgE) levels and asthma control status.

Current evidence demonstrates that IgE is mostly produced locally in patients with respiratory allergy.^13^ Measurement of IgE in local tissue could be useful in monitoring disease status and treatment response. Local IgE measurement in induced sputum is a safe, noninvasive, and feasible procedure that can be performed in both adults and children. This study aimed to investigate the predictive values of pulmonary inflammation indicators including pulmonary function test indices, FeNO, and induced sputum IgE for levels of asthma control.

## 2 METHODS

### 2.1 Study design and patients

This is a cross‐sectional study involving 117 patients with asthma continuously from January 1st 2022 to January 31st 2024. All patients were aged from 18 to 70 without gender restrictions. Asthma was diagnosed by respiratory physicians according to the GINA 2022 criteria. Patients required at least one typical symptom, including wheezing, shortness of breath, chest tightness, or coughing. Additionally, participants needed to meet one objective test for variable expiratory airflow limitation, including (1) positive bronchodilator responsiveness test: 15 min after inhaling 200–400 μg of salbutamol, an increase in FEV1 of 12% and more than 200 mL from baseline indicated a positive bronchodilator response; (2) positive exercise challenge test: after inhaling hypertonic saline, fall in FEV1 of ≥10% and more than 200 mL indicated a positive exercise challenge test; (3) excessive variation in lung function between visits: a change in FEV1 of >12% and more than 200 mL between visits indicated excessive variation; (4) improvement in lung function after 4 weeks of ICS treatment: an increase in FEV1 of >12% and more than 200 mL from baseline indicated significant improvement.

Patients were excluded from the study if they met any of the following criteria: (1) Received systemic steroid therapy in the proceeding 4 weeks; (2) received immunosuppressive therapy or biologics treatment; (3) diagnosed with other lung diseases such as chronic obstructive pulmonary disease, pneumonia, active pulmonary tuberculosis; and (4) diagnosed with congenital or acquired immunodeficiency, lupus, severe heart failure or cancer. We also included 10 subjects as the control group that had no upper and lower airway inflammatory diseases or other systemic diseases. This study was approved by the medical ethics committee of Beijing Tongren Hospital, Capital Medical University.

Demographic and clinical data were collected from each patient, including demographic characteristics (age, gender, body mass index (BMI), current smoker, and atopy), peripheral blood examination (blood cytology, and serum IgE), standard questionnaires (Asthma Control Test (ACT) score, Sino‐Nasal Outcome Test‐22 (SNOT‐22) score, Rhinoconjunctivitis Quality of Life Questionnaire (RQLQ) score), FeNO, and pulmonary function test.

### 2.2 Definition of asthma control

Asthma control was assessed by clinicians at the time of registration using the asthma control assessment questions from the GINA 2022 guidelines. Patients with asthma were categorized as WCA, PCA, and UCA based on 4 characteristics in the past 4 weeks: daytime asthma symptoms more than 2 times per week (yes or no), night waking due to asthma (yes or no), the need for SABA reliever medication for symptoms more than 2 times per week (yes or no), and limitation of activities due to asthma (yes or no). We assigned a score of 1 point to each response with “yes”, and 0 point for “no”. Patients in the WCA group had none of these asthma characteristics (scoring 0 points), while individuals with PCA had 1 or 2 of these characteristics (scoring 1 to 2 points), and individuals with UCA had 3 or 4 of these characteristics (scoring 3 to 4 points).

### 2.3 Sputum induction and processing

Sputum induction was performed using inhalation of hypertonic saline (4.5%) through a mouthpiece connected to an ultrasonic nebulizer (PARI BOY N 085, PARI GmbH, Germany) with maximum output settings for periods of 10 min. Induction was stopped if any of the following criteria was met: (1) The patient produced an adequate sputum sample, (2) no sputum was produced after 30 min of induction, and (3) PEF dropped below 80% of the baseline value. The sputum samples were kept on ice and processed within 2 h. The percentage of sputum eosinophils (Eos%) was obtained by counting 400 non‐squamous cells. The sputum cell viability was determined using Acridine Orange/Propidium Iodide (AO/PI) and analyzed using a fluorescence microscope. Sputum was weighed and mixed with 0.1% dithiothreitol in a ratio of 1:1. All samples were filtered through a 48‐μm gauze to increase homogenization and centrifuged at 1500 g for 10 min at 4°C. The supernatants from the sputum samples were harvested and stored at −80°C for further analysis.

### 2.4 Pulmonary evaluation and sputum IgE measurement

Pulmonary inflammation indicators, including FeNO, pulmonary function, and sputum IgE, were evaluated. A nitric oxide analyzer (Niox; Aerocrine) was used to measure FeNO at a flow rate of 50 mL/s via the oral cavity, and pulmonary function was measured by Spirometry (JAEGER, MasterScreen‐body + diffusion + APS). Levels of total IgE and IgE to a well‐balanced mixture of common inhalant allergens (Phadiatop test) in induced sputum were measured using an automatic immunoassay system (Phadia 1000, Thermo Fisher Scientific, America) according to the manufacturer's instructions. In addition, supernatants from sputum samples were assayed for the levels of total IgE using ELISA kits (Arigo, ARG80140) according to the manufacturer's instructions.

### 2.5 Logistic regression and ROC curve

Parallel line test was performed on factors with significant differences between three groups of asthma, including BMI, FEV1/estimated value of FEV1 (FEV1%pred), FEV1/forced vital capacity (FEV1/FVC), maximal expiratory flow at 25%, 50%, 75% and 75/25 of the FVC (MEF75, MEF50, MEF25 and MEF75/25 respectively), FeNO, blood eosinophil percentage, blood eosinophil counts, ACT score, SNOT‐22 score. RQLQ score, sputum total IgE, sputum Phadiatop, and serum total IgE. Factors with *P* < 0.05 failed the parallel line assumption and therefore were screened out. A univariate ordinal logistic model was then used to test the relationship between asthma control levels and pulmonary inflammation indicators. Factors with the largest variance inflation in the model testing by collinearity diagnostics were excluded, and finally nine factors were used for multifactor ordinal logistic regression analysis. A *p* value < 0.05 was considered significant. Receiver operating characteristic curve (ROC) analysis was used to evaluate the diagnostic accuracy of sputum total IgE in identifying UCA and WCA in asthmatic patients.

### 2.6 Statistical analysis

Data generated in this study were analyzed using GraphPad Prism 9 (GraphPad Software, Inc., USA) and SPSS version 27.0 (IBM Corp.). Demographic and clinical characteristics of the participants were summarized using means and standard deviations for continuous variables, and counts and percentages for categorical variables. The variables between groups were compared by one‐way analysis of variance or Chi‐square test. Correlation analysis was performed using Spearman's correlation. Outliers in the dataset were removed according to the “three sigma rule”.^14^ A *p* value of <0.05 was considered statistically significant.

## 3 RESULTS

### 3.1 Demographic and clinical characteristics of study participants

Demographic and clinical characteristics of three groups of patients with asthma (WCA, PCA, and UCA) and control subjects are presented in Table 1 and Table S1 in Supporting Information S1. The control group had significantly lower levels of sputum total IgE and Phadiatop, and better ACT, SNOT‐22 and RQLQ scores compared with three groups of asthma (*p* < 0.05, respectively; Table S1). Also, the control group had lower levels of blood eosinophils and FeNO compared with both PCA and UCA group (*p* < 0.05, respectively; Table S1). There was no significant difference regarding age, gender, current smoker, comorbid allergic diseases, and blood lymphocytes, monocytes, neutrophils and basophils among the three groups of asthma control. Significant differences in BMI, peripheral blood eosinophils, serum total IgE, ACT scores, RQLQ scores and SNOT‐22 scores were found among three groups. Patients with UCA have significantly higher BMI compared with PCA group (*P* < 0.001) and significantly higher blood eosinophils (percentage and count number; *P* < 0.001 respectively) and serum total IgE (*P* < 0.001) compared with the WCA group. Furthermore, the UCA group also had the highest SNOT‐22 score and RQLQ score among the groups (*P* < 0.05, respectively), and lower ACT score compared with PCA (*P* = 0.005) and WCA (*P* < 0.001) groups. Additionally, patients with PCA had a lower ACT score (*P* = 0.025) and higher blood eosinophils (*P* = 0.007) than the WCA group.

**TABLE 1:** Characteristics of subjects enrolled in this study.

Characteristics	WCA	PCA	UCA	*p* value
	(*n* = 40)	(*n* = 37)	(*n* = 40)	
Age, year	43.1 ± 13.17	42.57 ± 14.56	42.8 ± 13.93	0.986
Male, *n* (%)	17 (42.5)	11 (29.7)	17 (42.5)	0.418
BMI, kg/m^2^	24.86 ± 4.58	24.08 ± 3.55	26.72 ± 4.99	0.03*
Current smoker, *n* (%)	5 (12.5)	7 (19.4)	13 (32.5)	0.084
With other allergic diseases, *n* (%)	13 (32.5)	13 (35.1)	22 (55)	0.084
Blood lym, %	30.91 ± 7.97	31.2 ± 8.54	28.6 ± 8.95	0.381
Blood mon, %	5.94 ± 1.49	5.6 ± 1.22	5.49 ± 1.72	0.409
Blood bas, %	0.59 ± 0.26	0.7 ± 0.38	0.66 ± 0.3	0.33
Blood eos, %	3.29 ± 3.23	5 ± 3.43	7.4 ± 6.57	0.002*
Blood neu, %	59.28 ± 8.65	57.5 ± 10.25	57.85 ± 11.78	0.746
Blood lym, 10^9^/L	1.91 ± 0.53	1.94 ± 0.66	2.06 ± 0.6	0.544
Blood mon, 10^9^/L	0.38 ± 0.12	0.35 ± 0.11	0.4 ± 0.14	0.29
Blood bas, 10^9^/L	0.04 ± 0.02	0.05 ± 0.03	0.05 ± 0.03	0.128
Blood eos, 10^9^/L	0.22 ± 0.26	0.36 ± 0.33	0.56 ± 0.54	<0.001*
Blood neu, 10^9^/L	3.78 ± 1.06	3.71 ± 1.48	4.38 ± 1.59	0.09
ACT score	22.33 ± 2.41	17.86 ± 3.81	15.19 ± 3.73	<0.001*
SNOT‐22 score	17.42 ± 12.83	21.03 ± 14.98	32.11 ± 18.33	<0.001*
RQLQ score	16.92 ± 20.37	24.57 ± 21.83	44.34 ± 25.39	0.001*

*Note: p* value represents the difference between three groups, which compared by one‐way analysis of variance or Chi‐square test.

*Statistical significance.

Abbreviations: ACT, asthma control test; BMI, body mass index; PCA, partly controlled asthma; RQLQ, rhinoconjunctivitis quality of life questionnaire; SNOT‐22, Sino‐Nasal Outcome Test‐22; UCA, uncontrolled asthma; WCA, well controlled asthma.

### 3.2 Pulmonary inflammation indicators differ in patients with different levels of asthma control

**FIGURE 1:**
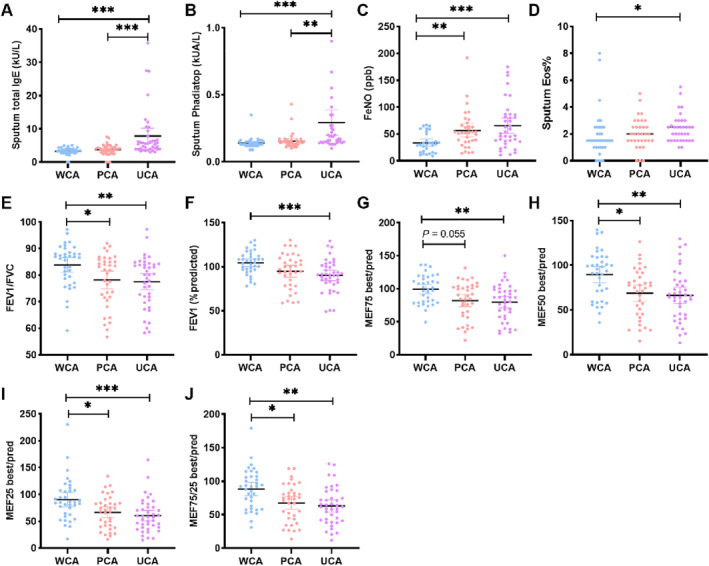
Pulmonary inflammation indicators in patients with different levels of asthma control. (A–B) Levels of total local immunoglobulin E (IgE) and IgE to a well‐balanced mixture of common inhalant allergens (Phadiatop test) in induced sputum. (C) Measurement of fractional exhaled nitric oxide (FeNO). (D) Measurement of eosinophil percentage (Eos%) in induced sputum. (E–J) Indicators of pulmonary function. *p < 0.05, **p < 0.01, ***p < 0.001. PCA, partly controlled asthma; UCA, uncontrolled asthma; WCA, well controlled asthma.

The induced sputum samples were collected and harvested for cells and supernatants, respectively. Cell viability analysis showed that the percentage of viability was 57.7% ± 7.6% (Figure S1). Supernatants were used for the measurement of total IgE and Phadiatop test. The total IgE concentration measured by ImmunoCAP and ELISA were highly correlated (*r* = 0.81, *p* < 0.001; Figure S2), indicating high reliability of ImmunoCAP for detection of sputum IgE. Patients with UCA have significantly higher levels of sputum total IgE and sputum Phadiatop (Figure 1A–B, *P* < 0.01 respectively) compared with both PCA and WCA groups, and higher levels of FeNO (Figure 1C, *P* < 0.001) and sputum eosinophil percentage (Eos%) (Figure 1D, *P* < 0.05) compared with the WCA group. On the contrary, indices of pulmonary function including FEV1%pred, FEV1/FVC, MEF75, MEF50, MEF25, and MEF75/25 were significantly lower in the UCA group than in the WCA group (Figure 1E–J, *P* < 0.01 respectively). In addition, patients with PCA had higher levels of FeNO (Figure 1C, *P* = 0.007) compared with the WCA group. FEV1/FVC (*P* = 0.028), MEF50 (*P* = 0.015), MEF25 (*P* = 0.019) and MEF75/25 (*P* = 0.024) were significantly lower in PCA group than in the WCA group (Figure 1E,H–J).

### 3.3 Sputum total IgE levels reflect asthma control status

To identify independent risk factors related to asthma control, variables that have significant differences between three groups of asthma control including basic characteristics (BMI, SNOT22 score, and RQLQ score), blood examination (blood eosinophil%, blood eosinophil counts, and serum total IgE), and pulmonary indicators (sputum total IgE, sputum Phadiatop, sputum Eos%, FeNO, FEV1%pred, FEV1/FVC, MEF75, MEF50, MEF25, and MEF75/25) were performed with ordinal logistic regression analysis. Univariate ordinal logistic regression analysis showed that FEV1%pred, FEV1/FVC, MEF75, MEF50, MEF25, MEF75/25, blood eosinophil%, blood eosinophil counts, sputum total IgE, sputum Phadiatop, serum total IgE, SNOT22 score and RQLQ score were potential risk factors. After removing collinear variables, nine items were entered into a multifactor ordinal logistic regression analysis. The results showed that sputum total IgE (OR = 6.25, 95% CI, 1.07–36.55) was the unique risk factor of asthma control status (Table 2).

**TABLE 2:** Ordinal logistic regression analysis of factors with significant differences between three groups of asthma control.

Variables	Parallel line test	Ordinal logistic regression			Multifactor ordinal logistic regression		
	*p* value	OR	(95% CI)	*p* value	OR	(95% CI)	*p* value
BMI, kg/m^2^	0.010	—	—	—			
FEV1%pred	0.339	0.55	0.38–0.80	0.002	1.14	0.54–2.41	0.737
FEV1/FVC	0.109	0.60	0.42–0.87	0.006	0.89	0.40–2.00	0.779
MEF75best/pred	0.101	0.58	0.40–0.83	0.003^†^			
MEF50best/pred	0.082	0.53	0.37–0.77	<0.001^†^			
MEF25best/pred	0.280	0.48	0.32–0.72	<0.001	0.60	0.24–1.52	0.283
MEF75/25best/pred	0.173	0.50	0.34–0.74	<0.001^†^			
FeNO,ppb	0.017	—	—	—			
Blood eos,%	0.509	2.37	0.45–3.88	<0.001	1.06	0.58–1.76	0.854
Blood eos,10^9^/L	0.491	2.24	1.37–3.67	0.001^†^			
SNOT‐22 score	0.641	2.12	1.39–3.22	<0.001	1.50	0.75–3.00	0.252
RQLQ score	0.995	2.34	1.51–3.62	<0.001	1.51	0.74–3.09	0.258
ACT score	0.019	—	—	—			
Sputum total IgE, kU/L	0.284	17.05	4.65–62.47	<0.001	6.25	1.07–36.55	0.042*
Sputum phadiatop, kUA/L	0.873	10.07	2.53–39.97	0.001	1.58	0.40–6.21	0.511
Serum total IgE, kU/L	0.246	2.00	1.16–3.44	0.012	0.88	0.44–1.74	0.706
Sputum eos%	0.983	1.35	0.93–1.94	0.112			

*Note:* Parallel line test was performed in factors with significant difference between three groups of asthma, and subsequently conducted univariate ordinal logistic regression. Factors with *p* < 0.05 failed the parallel line assumption and therefore were screened out. Factors (^†^) with the largest variance inflation in the model testing by collinearity diagnostics were excluded, and finally nine factors were used for multifactor ordinal logistic regression analysis.

*Significant risk factor.

**FIGURE 2:**
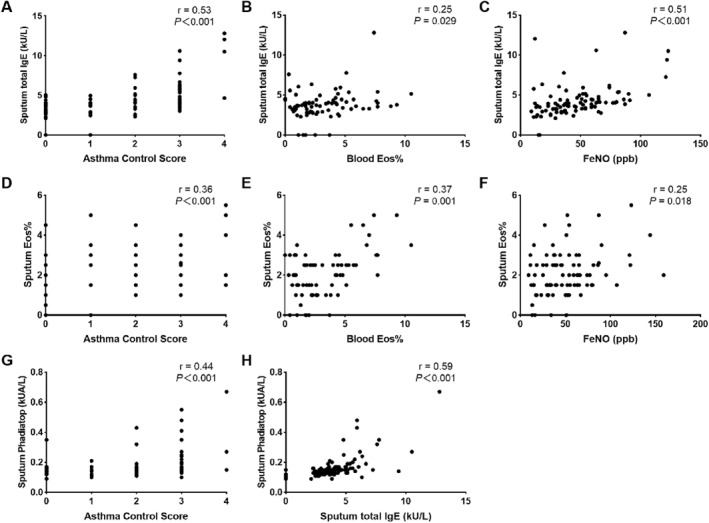
Correlations between sputum local immunoglobulin E (IgE), sputum eosinophils, and other inflammation indicators. (A–C) Correlations between sputum total IgE and asthma control score, blood Eos% and FeNO; (D–F) Correlations between sputum eosinophil percentage (Eos%) and asthma control score, blood Eos% and FeNO; (G) Correlations between sputum Phadiatop with asthma control scores; and (H) Correlation between sputum total IgE with sputum Phadiatop. Outliers were not presented in the chart.

Correlation analysis further indicated that sputum total IgE levels were significantly correlated with asthma control scores (Figure 2A; *r* = 0.53, *P* < 0.001), blood Eos% (Figure 2B; *r* = 0.25, *P* = 0.029), FeNO (Figure 2C; *r* = 0.51, *P* < 0.001), and sputum Phadiatop (Figure 2H; *r* = 0.59, *P* < 0.001). Although sputum Phadiatop was not a significant risk factor for asthma control, it showed a positive correlation with asthma control scores (Figure 2G; *r* = 0.44, *P* < 0.001). In addition, sputum Eos% was significantly correlated with asthma control score (*r* = 0.36, *P* < 0.001), blood Eos% (*r* = 0.37, *P* = 0.001), and FeNO (*r* = 0.25, *P* = 0.018) (Figure 2D–F). However, ordinal logistic regression analysis indicated that sputum Eos% was not a predictor of asthma control outcomes (*P* = 0.112; Table 2).

**FIGURE 3:**
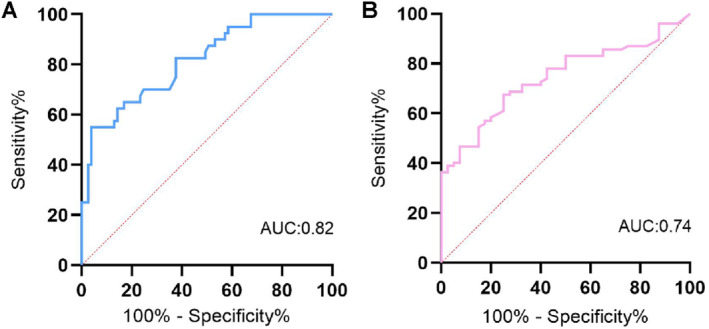
ROC analysis for the prediction of patients with uncontrolled asthma (UCA) (A) and WCA (B). AUC, area under the ROC; UCA, uncontrolled asthma; WCA, well controlled asthma.

To further assess the prognostic value of sputum total IgE for asthma control, ROC analysis was performed. Sputum total IgE showed high accuracy as a predictor of patients with UCA (Figure 3A, AUC = 0.82 (0.74–0.9)). When a cutoff value of >4.53 kU/L was set for total sputum IgE, the sensitivity and specificity for predicting UCA were 62.50% and 85.71%. In addition, sputum total IgE also showed a predictive value for WCA (Figure 3B, AUC = 0.74 (0.66–0.83)).

Additionally, to investigate whether the levels of sputum IgE are also associated with the severity of asthma, patients were divided into mild asthma (*n* = 84), moderate asthma (*n* = 16) and severe asthma (*n* = 12) according to the diagnostic criteria of the GINA guidelines. However, the levels of total IgE and Phadiatop in induced sputum had no significant difference among different groups of asthma severity (Figure S3).

## 4 DISCUSSION

Asthma symptom control directly impacts quality of life and health care resource use, which is the long‐term goal of asthma management.^15^ Patients suffering from severe and UCA are associated with substantial long‐term and short‐term consequences including limitations in physical and functional activities.^16^, ^17^ Evaluating asthma control status plays a crucial role in guiding asthma management strategies and assessing future risks. In real‐world practice, clinicians need more indicators not limited to simple patient‐reported questionnaires such as ACT and ACQ scores to assist with the assessment of asthma control. The present study demonstrates significant differences in pulmonary inflammation indicators among different levels of asthma control, and identified sputum total IgE as the only independent risk factor for patients with UCA. Those findings demonstrate that levels of total IgE in induced sputum reflect asthma control status, which could be an important clinical indicator for the assessment of asthma control.

Pulmonary indicators are closely related to the severity of asthma symptoms. Pulmonary function tests can quantify the airflow limitation, which reflects symptom severity, quality of life and clinical outcomes.^17^, ^18^ However, studies have found that indices of pulmonary function are not significantly correlated with levels of asthma control.^19^ There have been some findings indicated that peripheral airway impairment was significantly associated with UCA. Impulse Oscillometry, which can evaluate the obstruction of small airways and detect airway changes earlier than standard spirometry criteria, has been suggested for assessing asthma control in children. However, IOS lacks the ability to classify patients correctly according to the status of asthma control in adult patients.^20^, ^21^, ^22^, ^23^

Sputum eosinophil levels are considered the gold standard for identifying type 2 inflammatory asthma, and are associated with asthma severity, exacerbations, and response to corticosteroid therapy.^24^, ^25^, ^26^ To explore the relationship between sputum eosinophils and asthma control levels, Demarche et al. conducted a retrospective longitudinal study involving 187 patients, and demonstrated that asthma control was associated with sputum eosinophil counts fluctuating over time at the individual level.^27^ However, Volbeda et al. reported that the levels of asthma control were not significantly associated with sputum eosinophil levels, despite patients with UCA having significantly more sputum eosinophils.^24^ Similarly, a recent cross‐sectional study in a population with severe asthma illustrated that eosinophilic sputum (sputum eosinophils ≥3%) was not related to asthma control.^28^ Our findings also indicated that sputum Eos% was not a predictor of asthma controls. In addition, FeNO is a surrogate biomarker for the assessment of severe asthma and prediction of responsiveness to some biological therapies.^29^, ^30^, ^31^ Saito et al. demonstrated that absolute levels of FeNO are not associated with asthma control.^8^ Moreover, FeNO showed poor accuracy in the differentiation of well‐controlled asthma and not well‐controlled asthma in children.^5^ Correspondingly, the present study showed that patients with UCA have significantly higher levels of FeNO than WCA, however, FeNO is not a significant risk factor for asthma control.

Induced sputum has been widely acknowledged as a safe, noninvasive, reproducible examination for the assessment of airway inflammation in both adults and children, which is substantially useful in routine clinical practice.^32^, ^33^ The sputum supernatant is well suited for measuring various inflammatory indicators including cytokines, chemokines, granulocyte proteins, and vascular leakage markers. Previous studies have demonstrated that IgE measurement in induced sputum was valid using a commercially available immunoassay. Additionally, the use of dithiothreitol reagent during induced sputum processing did not affect IgE quantification.^34^ In view of accumulating evidence that local IgE in nasal secretions showed diagnostic value for allergic rhinitis,^35^, ^36^ this study investigated the association between sputum IgE levels and asthma control. Total IgE and IgE of common inhalant allergens in both induced sputum and serum were detected using an ImmunoCAP instrument. We found that sputum total IgE levels were significantly correlated with asthma control scores. Although serum total IgE was also correlated with asthma control scores, the correlation seems much weaker (Figure S4). Due to the fact that IgE in peripheral blood could originate from various organs that suffer from allergen exposure, its levels may lack accuracy in assessing asthma control status or local type 2 inflammation. Our findings suggest that sputum IgE levels may better reflect the pulmonary inflammation status.

Additionally, the present study also indicates other indices that significantly differentiated in different levels of asthma control, such as BMI, pulmonary function, FeNO, blood eosinophil, as well as SNOT‐22 score and RQLQ score related to nasal inflammation, which are consistent with previous findings.^27^, ^37^, ^38^ However, sputum total IgE is a unique significant risk factor for poor asthma control.

Accumulating evidence indicated that local IgE production has been identified in various samples from asthma patients, such as bronchiolar lavage fluid, induced sputum, and lung tissue sections.^25^, ^26^, ^28^, ^39^ Balzar et al. performed immunostaining for IgE in lung tissue sections from asthma patients, and concluded that the identification of local IgE in patients was associated with more severe asthma exacerbations.^29^, ^40^ Similarly, Schleich et al. found that patients with asthma only sensitized to *staphylococcus aureus* enterotoxins have more exacerbations, and higher levels of sputum IgE.^41^ It has also been reported that levels of total IgE and specific IgE to dermatophagoides pteronyssinus in the sputum are increased in patients with intrinsic asthma compared to non‐atopic individuals.^42^ Furthermore, sputum total IgE may also serve as a potential biomarker for guiding anti‐IL‐5 therapy in patients with severe asthma, with higher levels of sputum total IgE possibly predicting better responses to anti‐IL‐5 therapy.^43^ Thus, those findings indicate that the measurement of sputum IgE is necessary for monitoring asthma exacerbations, identifying specific phenotype of asthma, and guiding treatment.

This study is somewhat limited. First, the sample size is relatively small. A large sample size is necessary for future studies to confirm the clinical values of sputum total IgE in reflecting asthma control levels and predicting UCA. Second, the sensitivity for detecting sputum IgE should be further improved with current measurement techniques. In this study, levels of IgE were measured using an automatic immunoassay ImmunoCAP Phadia system, which is the gold standard for measuring the amount of circulating IgE in human serum or plasma samples. However, the calibrator reagents and curve controls of IgE were designed for serum or plasma samples, which may not be the optimal condition for sputum samples. It is necessary to optimize the calibrator curve to improve the sensitivity for detecting sputum IgE.

Taken together, our findings demonstrate that sputum total IgE is generally high in patients with UCA, and correlates with asthma control status, which can be used as an indicator for UCA.

## AUTHOR CONTRIBUTIONS

**Wenhui Chen**: Methodology; writing—original draft; writing—review and editing; investigation. **Xiaofang Liu**: Methodology; investigation; writing—review and editing. **Xiujuan Yao**: Methodology; investigation. **Yanghe Hao**: Investigation. **Zhuo Zhou**: Investigation. **Chengshuo Wang**: Writing—review and editing; funding acquisition. **Ming Wang**: Conceptualization; methodology; investigation; writing—original draft; writing—review and editing; funding acquisition; project administration. **Luo Zhang**: Conceptualization; methodology; investigation; funding acquisition; project administration; writing—review and editing; writing—original draft.

## CONFLICT OF INTEREST STATEMENT

The authors declare that they have no conflicts of interest.

## Supporting information

Supporting Information S1

## Data Availability

The data that support the findings of this study are available from the corresponding author upon reasonable request.
