# Supplementary material for: Total immunoglobulin E levels in induced sputum reflect asthma control status
Source: Clin Transl Allergy. 2025 Jan 5;15(1):e70021. doi: 10.1002/clt2.70021 (PMC11702434; doi:10.1002/clt2.70021)

**Supplementary tables**

**Table S1. Characteristics of control subjects enrolled in this study**

| **Characteristics** | **Control**  **(n=10)** | ***P* value** | ***P* value** | ***P* value** |
| --- | --- | --- | --- | --- |
|  |  | **(WCA vs. Control)** | **(PCA vs. Control)** | **(UCA vs. Control)** |
| Age, year | 37.11±9.64 | 0.134 | 0.307 | 0.148 |
| Male, n (%) | 3（30） | 0.719 | 0.999 | 0.999 |
| BMI, kg/m^2^ | 24.94±4.56 | 0.832 | 0.435 | 0.430 |
| Current smoker, n (%) | 1（10） | 0.999 | 0.999 | 0.653 |
| With other allergic diseases, n (%) | 0（0） | 0.084 | 0.044* | 0.002* |
| Blood Lym, % | 31.13±10.70 | 0.759 | 0.110 | 0.094 |
| Blood Mon, % | 7.16±2.49 | 0.246 | 0.805 | 0.956 |
| Blood Bas, % | 0.53±0.39 | 0.241 | 0.235 | 0.162 |
| Blood Eos, % | 1.56±1.73 | 0.162 | 0.005* | 0.015* |
| Blood Neu, % | 59.63±11.82 | 0.556 | 0.232 | 0.432 |
| Blood Lym, 10^9^/L | 1.92±0.69 | 0.882 | 0.790 | 0.394 |
| Blood Mon, 10^9^/L | 0.44±0.15 | 0.284 | 0.125 | 0.651 |
| Blood Bas, 10^9^/L | 0.03±0.03 | 0.217 | 0.196 | 0.049* |
| Blood Eos, 10^9^/L | 0.10±0.12 | 0.203 | 0.009* | 0.009* |
| Blood Neu, 10^9^/L | 3.71±1.05 | 0.923 | 0.717 | 0.277 |
| ACT score | 24.56±1.01 | ＜0.001* | ＜0.001* | ＜0.001* |
| SNOT-22 score | 2.78±3.42 | 0.034* | ＜0.001* | ＜0.001* |
| RQLQ score | 1.12±1.25 | ＜0.001* | ＜0.001* | ＜0.001* |
| Sputum total IgE，kU/L | 1.93±1.41 | ＜0.001* | 0.002* | ＜0.001* |
| Sputum Phadiatop，kUA/L | 0.13±0.12 | 0.003* | ＜0.001* | ＜0.001* |
| FeNO, ppb | 25.70±22.15 | 0.110 | 0.001* | 0.001* |

***P*** value represents the difference between two groups, which compared by Mann-Whitney U test or χ2 test. * Statistical significance. WCA, well controlled asthma; PCA, partly controlled asthma; UCA, uncontrolled asthma; BMI, body mass index; ACT, asthma control test; SNOT-22, Sino-Nasal Outcome Test-22; RQLQ, rhinoconjunctivitis quality of life questionnaire; FeNO, fractional exhaled nitric oxide.

**Supplementary figures**

**Figure S1.** **Cell viability analysis of induced sputum using Acridine Orange/Propidium Iodide (AO/PI).** (A) all nucleated cells stained with AO (green). (B) Dead cells stained with PI (red). (C) Bright-field and combined images of AO/PI staining. (D) proportion of living and dead cells in sputum.

**
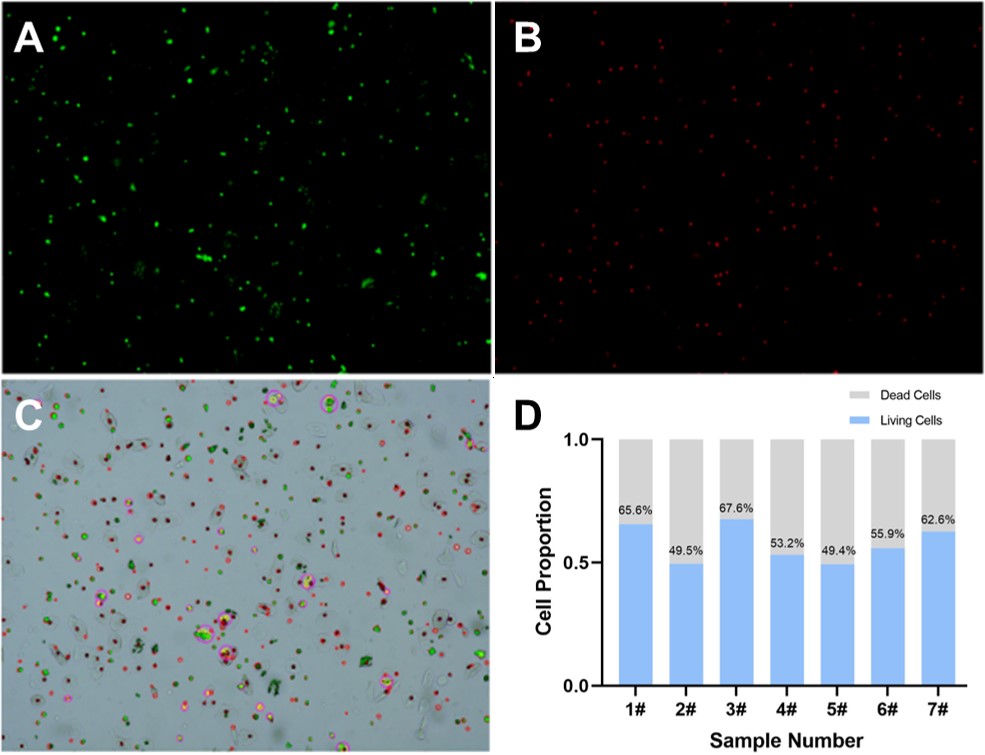
**

**Figure S2. Correlations of ELISA and ImmunoCAP in determining sputum total IgE.** Scatter plot of the correlation analysis between total IgE concentrations measured by ImmunoCAP and ELISA.


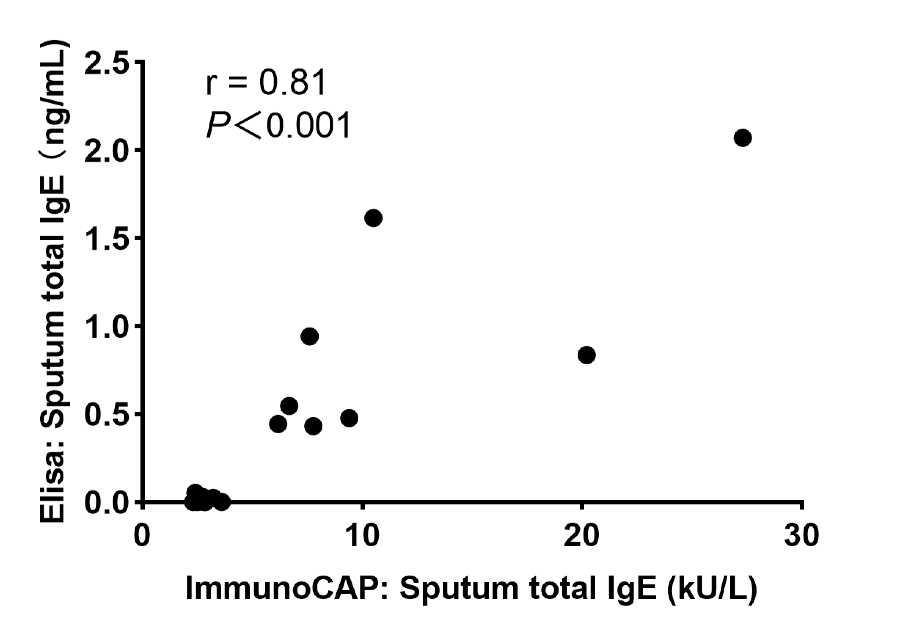


**Figure S3. Sputum total IgE and phadiatop in patients with different levels of asthma severity.** Levels of total IgE (A) and IgE to a well-balanced mixture of common inhalant allergens (Phadiatop test) (B) in induced sputum among patients with different levels of asthma severity (mild, moderate, and severe asthma).


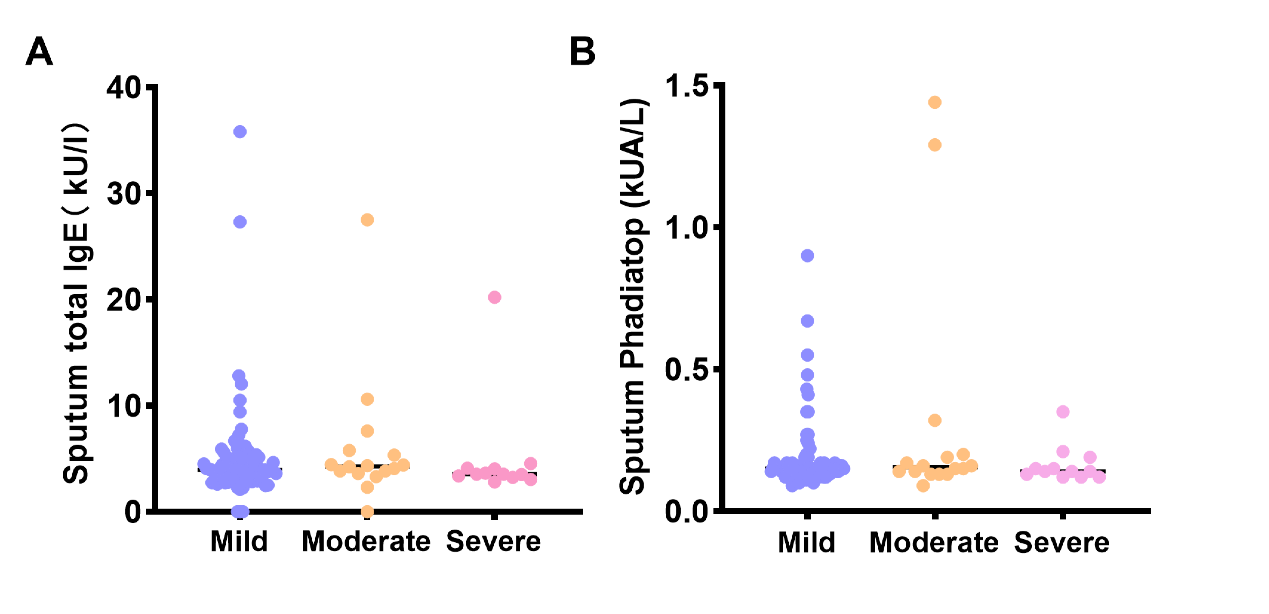


**Figure S4. Correlations between serum total IgE and asthma control score.** Scatter plot of the correlation analysis between serum total IgE concentrations and asthma control scores.


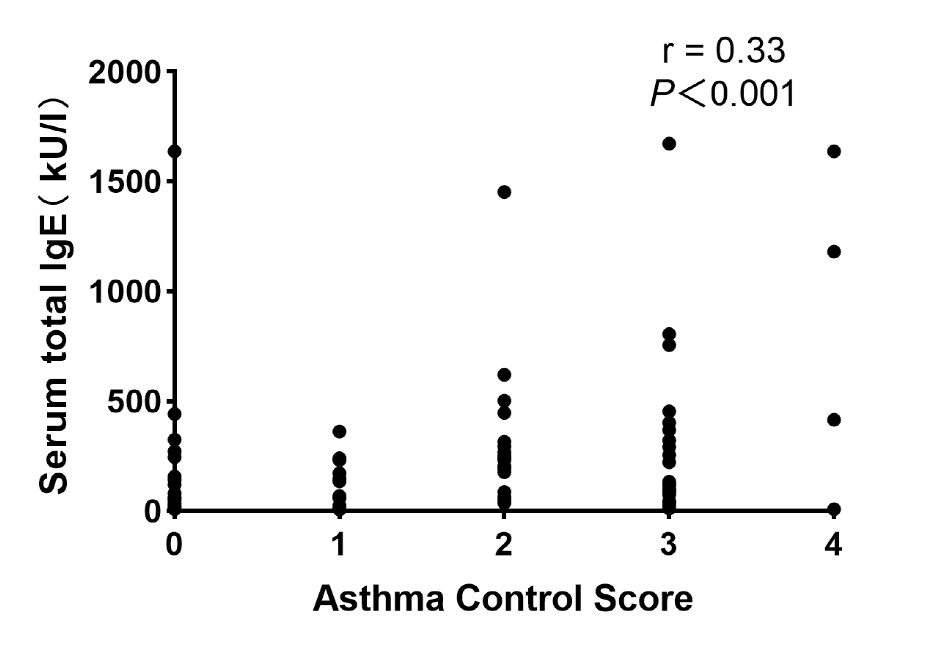

Supplement: Supplementary file 1 — Supporting Information S1 [file CLT2-15-e70021-s001.docx]
